# Contributions of distinct attention mechanisms to saccadic choices in a gamified, dynamic environment

**DOI:** 10.1101/2025.01.25.634882

**Published:** 2025-01-26

**Authors:** Evan A Kattner, Terrence R Stanford, Emilio Salinas

**Affiliations:** Department of Translational Neuroscience, Wake Forest University School of Medicine, 1 Medical Center Blvd., Winston-Salem, NC 27157-1010, USA

## Abstract

Visuospatial attention is key for parsing visual information and selecting targets to look at. In turn, three types of mechanism determine when and where attention is deployed: stimulus-driven (exogenous), goal-driven (endogenous), and history-driven (reflecting recent experience). It is unclear, however, how these distinct attentional signals interact and contribute during natural visual scanning, when stimuli may change rapidly and no fixation requirements are imposed. Here, we investigate this via a gamified task in which participants make continuous saccadic choices at a rapid pace — and yet, perceptual performance can be accurately tracked over time as the choice process unfolds. The results reveal unequivocal markers of exogenous capture toward salient stimuli; endogenous guidance toward valuable targets and relevant locations; and history-driven effects, which produce large, involuntary modulations in processing capacity. Under dynamic conditions, success probability is dictated by temporally precise interplay between different forms of spatial attention, with recent history making a particularly prominent contribution.

**Significance Statement:**

Visuospatial attention comprises a collection of mental mechanisms that allow us to focus on (or look at) specific objects or parts of space and ignore others. The next target to be inspected is generally selected based on how much it stands out (salience), its relevance to current goals, and recent experience. We designed a gamified visual scanning task in which all such forms of attentional control interact rapidly, more akin to real life situations (e.g., driving through traffic). Each mechanism affected in characteristic ways the probability that participants would look to the correct target at each moment in time. Most notably, we found that the history of recently seen stimuli determines visual processing capacity much more strongly than previously thought.

## Introduction

Day-to-day we experience a complex visual world, and navigating it successfully requires a system by which to prioritize information and select relevant parts. Visuospatial attention is the cognitive capacity to do precisely that, i.e., direct processing resources to areas of visual space that are important while ignoring areas that are not.

Much is known about the specialized mechanisms that enable the deployment of attention, either overtly (via eye movements) or covertly (with gaze fixed), to specific points in space (Itti and Koch, 2001; Corbetta and Shulman, 2002; Theeuwes, 2010; Carrasco, 2011; Wolfe and Horowitz, 2017). Bottom-up or exogenous mechanisms draw attention rapidly, transiently, and involuntarily toward physically salient stimuli. In contrast, top-down or endogenous mechanisms are slower and require effort, but permit the voluntary and sustained deployment of attention to potentially goal-relevant objects or locations regardless of physical salience. In addition to this traditional dichotomy, it is now widely recognized that recent experience also plays a critical role in determining the effective strength of visual features or locations (Fecteau and Munoz, 2003; Awh et al., 2012; Theeuwes, 2019; Anderson et al., 2021). Lingering biases due to prior history take many forms, depending on past stimuli, past actions, past rewards, etc (Maljkovic and Nakayama, 1994; Failing and Theeuwes, 2018; Jiang and Sisk, 2019; Kim and Anderson, 2019; Anderson et al., 2021). In primates, whenever oculomotor circuits redirect the eyes to a new fixation target (every 200–250 ms), the underlying selection process is heavily steered by the three types of mechanism just listed: stimulus-driven, goal-driven, and history-driven (Maunsell, 2015; Moore and Zirnsak, 2017; Westerberg and Schall, 2021).

Although these mechanisms have been studied extensively, prior work has largely relied on segmented laboratory tasks with prolonged fixation requirements. Thus, it is unclear what their contributions would be like under more dynamic and less constrained viewing conditions; say, while playing basketball or driving through traffic. Here, we investigate visuospatial attention in a laboratory experiment that takes a few steps toward such richness. Why is this important? More ecologically realistic situations may reveal not only subtle aspects of behavior that might easily go unnoticed, but also large, unexpected effects on visual processing (Camerer and Mobbs, 2017; Snow and Culham, 2021).

With more realism, quantifying performance becomes more challenging. To strike a balance, we designed a new paradigm that combines two elements, richer dynamics and so called “urgent” choice tasks (Stanford and Salinas, 2021). Urgent tasks impose time presssure to yield a more variable and realistic interaction between motor plans and perceptual information than traditional (non-urgent) tasks. The upshot is that it becomes possible to construct a unique psychophysical curve describing how a choice is elaborated as a function of time — and such “tachometric” curve reveals clear signatures of exogenous, endogenous, and history-based contributions to performance (Salinas et al., 2019; Goldstein et al., 2022, 2024; Oor et al., 2023, 2024)

Thus, we developed SpotChase, a gamified task that, rather than discrete trials, presents a continuous stream of choices akin to pro- and antisaccades. Instead of fixating and responding based on a rigidly timed script, participants make a series of perceptual judgements and eye movements at a (fast) pace that they control, aiming to obtain a high score across numerous choices in accordance with simple rules for accruing points. This produces scanning behavior that is highly interactive, closer to that of a basketball game or driving through traffic. Critically, though, SpotChase retains sufficient stimulus control to quantify visuomotor performance rigorously via tachometric curves.

Using SpotChase, we found unequivocal, temporally segregated behavioral signatures of exogenous and endogenous attention similar to those reported previously (Salinas et al., 2019; Goldstein et al., 2022, 2024; Oor et al., 2023). But in this case, selection history effects were most remarkable: they resulted in powerful, involuntary modulation of both the early (exogenously-dominated) and later (endogenously-dominated) phases of the target selection process, and accounted for wide differences in perceptual capacity between individual participants.

## Results

### SpotChase: a dynamic choice task

Participants completed “runs” of SpotChase, 90 second periods of continuous performance of choices akin to pro- and antisaccades, but guided by an overall goal of accruing as many points as possible by the end of each run.

**Figure 1.**
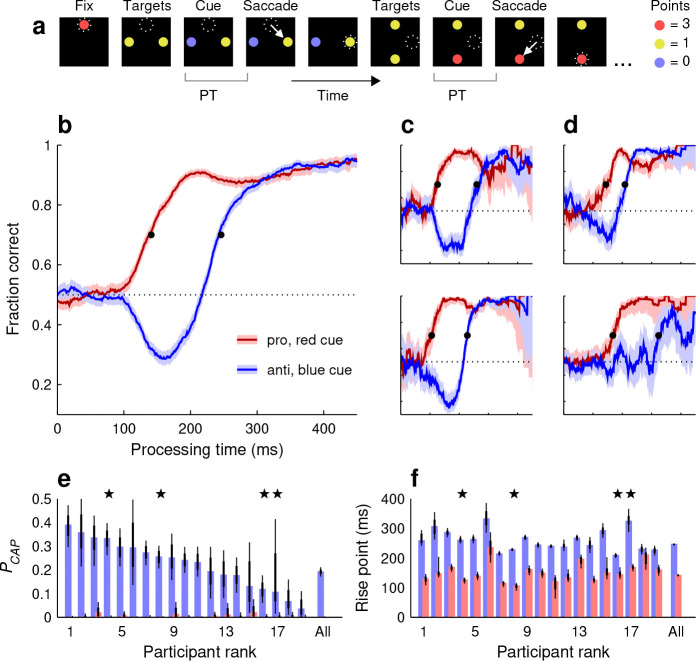
Performance in the base version of SpotChase. **a**, Sequence of events during the task. Each run begins with gaze (dotted circles) fixated on a red dot (Fix). This is followed by the appearance of two yellow targets aligned horizontally (Targets). After a variable time, one target switches to blue or red (Cue), prompting the participant to make a saccadic choice based on the point values shown. Once a choice is made (Saccade; arrows), vertical targets appear and the choice process starts anew. Thereafter, horizontal and vertical targets alternate until the run (90 s) ends. The processing time (PT) is the interval between cue onset and saccade onset. **b**, Tachometric curves for red-cue, or pro choices (red trace) and blue-cue, or anti choices (blue trace) combined across 19 participants. Shaded ribbons indicate 95% confidence intervals (CIs) across choices. Horizontal dotted line marks chance performance. Black dots indicate rise points. **c**, **d**, Tachometric curves from individual participants with high (**c**) or low (**d**) $P_{CAP}$ values. Same conventions as in **b**. Each curve from each participant is based on ~1,100 choices. **e**, Probability of capture ($P_{CAP}$) across participants. Bars indicate values for pro (red) and anti (blue) choices. Thin and thick lines indicate 95% and 68% CIs, respectively. Participants are sorted by their effect magnitude. Stars mark the example participants in **c** (pair on left) and **d** (pair on right). **f**, Curve rise points across participants. Same conventions and participant order as in **e**.

Every run began with an initial fixation on a red spot at the top-center of the screen (Fig. 1a, Fix). This was followed by presentation of two diametrically positioned yellow targets to the left and right of the center (Fig. 1a, Targets). After a variable interval (typically 50–300 ms), one of the two yellow targets switched to red or blue (Fig. 1a, Cue). After this, the display did not change until the participant made a choice by looking at one of the two colored spots (Fig. 1a, Saccade). Then, following a brief post-choice interval (150 ms), a new pair of yellow targets was presented, this time arranged vertically. This marked the start of a new choice cycle consisting of the same event sequence: Targets, Cue, and Saccade. Horizontal and vertical targets alternated throughout each run, allowing for seamless transitions between choices.

The participants aimed to accrue as many points as possible after each run, with points assigned according to the color of each chosen spot (Methods). Looking at a red, yellow, or blue target yielded 3, 1, or 0 points, respectively. In the base version of the task, red and blue cues were randomly interleaved with equal probability at all locations and had the same luminance as the yellow targets. Nevertheless, both cue stimuli were salient because of their sudden color onset. This, together with the scoring convention, established choice types similar to those of traditional prosaccade and antisaccade tasks, in that, to maximize their score, participants would direct their gaze towards a salient high-value (red) target or away from a salient low-value (blue) target.

It is worth emphasizing a few key differences between the continuous dynamic of SpotChase and traditional, trial-based oculomotor tasks. (1) After fixation on the red spot at the beginning of each run, no further fixation requirements were enforced. Participants learned to make choices efficiently, directing saccades mostly to the choice targets (Methods). (2) Feedback about performance was useful, but during data collection (after a few preliminary practice runs; Methods), it was provided only at the end of each run, in the form of a score. (3) The pace of the task was controlled by the participants, who were free to make choice saccades either before, shortly after, or well after each cue onset, increasing their probability of success accordingly. However, the longer they waited to respond, the fewer choices they could make. In order to maximize the score, it was best to go at a relatively fast pace at which guesses were frequent but more choices became available.

It is also important to note that SpotChase is similar to trial-based urgent tasks in one critical respect: for any given choice, it permits accurate measurement of the interval between cue onset and saccade onset, which is the maximum amount of time available for viewing and interpreting the cue. This quantity, which we call the processing time (**PT**), dictates performance in urgent tasks (Salinas et al., 2019; Poth, 2021; Stanford and Salinas, 2021; Goldstein et al., 2022, 2024; Oor et al., 2023, 2024), and we expected it would do so in SpotChase as well. The rationale is strightforward: the longer the PT, the higher the likelihood that the choice is guided by the cue information and, in turn, the greater the probability of choosing the higher-value target.

### SpotChase reveals signatures of exogenous and endogenous mechanisms

Nineteen participants performed the base version of Spotchase (Methods), on average generating 2.1 choices per second (range: 1.6–2.8 choices/s). Tachometric curves were used to quantify their performance (Methods). That is, choices were sorted into PT bins and, for each bin, the fraction of correct outcomes was calculated — where ‘correct’ means that the participant looked at the higher-value target on offer.

Separate tachometric curves were generated for red- and blue-cue choices (Fig. 1b–d). They revealed characteristic trends similar to those seen in urgent (trial-based) versions of the proand antisaccade tasks (Salinas et al., 2019; Goldstein et al., 2022). During short-PT choices (PT ≲ 90 ms), participants had little or no time to view the cue information and accuracy remained at chance level (50% correct) regardless of cue color. Responses in this range were uninformed and correspond to guesses. At the opposite extreme (PT & ≳ 300 ms), both curves achieved asymptotic accuracy levels near 90% correct, indicating that with sufficient cue viewing time participants consistently made informed choices in accordance with the goal of the task. Critically, in the transition between uninformed and informed choices, the salient cue onset gave rise to involuntary oculomotor capture, resulting in opposing deflections in performance for the two choice types. In blue-cue choices (Fig. 1b–d, blue traces), for which salience-driven and goal-driven signals pointed in opposite directions, performance first dropped well below chance (due to exogenous capture) before rising steadily (due to endogenous guidance). In contrast, in red-cue choices (Fig. 1b–d, red traces), the exogenous and endogenous signals were aligned, leading to a rapid, monotonic upswing in performance. These trends were evident for the pooled data (Fig. 1b) as well as for individual participants (Fig. 1c, d).

Two measures were used to quantify these effects. To characterize the strength of exogenous capture in anti (blue-cue) choices, we computed the probability of capture, or $P_{CAP}$ (Fig. 1e), which is the proportion of non-random responses that were incorrect (Methods). In essence, $P_{CAP}$ measures how much the tachometric curve dips below the chance line. This quantity was well above zero for the pooled data ($P_{CAP}=0.19$ in [0.17, 0.21], 95% CI, p<0.0001 from resampling test) and for 17 of the 19 individual participants ($P_{CAP}$ range was 0.09–0.38 for n=17 participants with p<0.0001;p>0.9 for n=2 participants; Fig. 1e, blue bars). By way of control, we computed $P_{CAP}$ for the pro (red-cue) tachometric curves, which in general rise steadily above 50% correct, and in this case none of the values were above those expected by chance (Fig. 1e, red bars). The exogenous capture toward the cue was highly robust, even though it acted briefly (for PTs in the 100–225 ms range, approximately) and even though the yellow, red, and blue spots were isoluminant (Methods).

In addition, to measure when the increase toward asymptotic accuracy occurred, we calculated the rise point of each tachometric curve (Fig. 1f), which was defined as the PT at which the fraction correct first exceeded 0.7 (Methods). By this measure, performance in pro choices rose much earlier (141 ms in [138, 145], 95% CI) than in anti choices (246 ms in [242, 250], 95% CI) based on the pooled data. The difference of 105 ms was consistent across individual participants, in that it was always positive (range: 20–197 ms, n=19) and statistically large (no overlap between 95% CIs for pro and anti rise points) in all but one of them (Fig. 1f). Thus, to produce a correct informed saccade, processing the anti (blue) cue typically required about 100 ms more than processing the pro (red) cue.

It is important to emphasize that the tachometric curves just discussed (Fig. 1b–d) quantify *perceptual* performance in SpotChase. To appreciate what this means, consider how urgency would impact the mean fraction correct over a full run. If the participants, say, increased their pace (rate of choices per second), one would expect a drop in fraction correct in accordance with the speed-accuracy tradeoff (Wickelgren, 1977; Chittka et al., 2009). By contrast, the tachometric curve is largely impervious to such tradeoff (Stanford et al., 2010; Salinas et al., 2014, 2019). If urgency increased, the left part of the curve would be sampled more heavily, so more guesses would be made and overall accuracy would drop — but its shape would not change.

**Figure 2.**
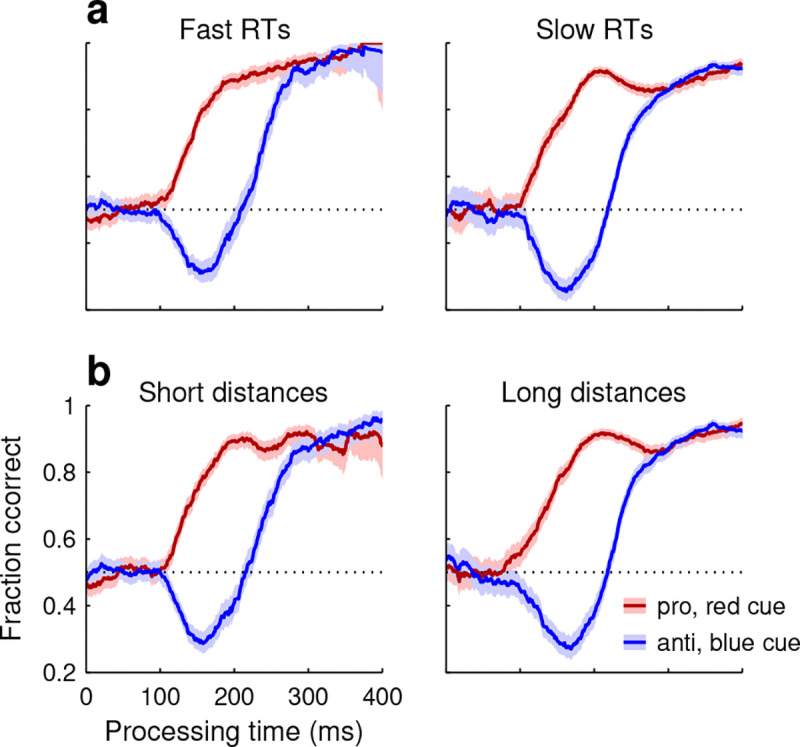
Performance in pro and anti choices depends weakly on urgency and task-specific features. **a**, For each participant, choices from the base version of SpotChase were split according to RT into fast (below the median RT) and slow (above the median RT). The data were then combined across participants. Tachometric curves are shown for the fast-RT (left) and slow-RT (right) data subsets, each subdivided into pro (red traces) and anti (blue traces) choices. Shaded ribbons denote 95% CIs. **b**, As in **a**, except that choices were split according to the total angular distance traveled by the eyes during each choice (from onset of targets to 100 ms after saccade onset).

To verify such decoupling of perceptual and motor processes in SpotChase, pro and anti tachometric curves were compared across urgency conditions. For each choice, the reaction time (**RT**) was measured from the onset of the yellow targets to the onset of the choice saccade (Methods). The data from each participant were then split by the median RT and aggregated across participants into two subsets, fast and slow. There were vast differences between the two resulting data subsets, not only in RT (for fast: 211 ± 88 ms, mean ± SD; for slow: 428 ± 148 ms) but also, as expected, in overall fraction correct (for fast: 0.55; for slow: 0.74). In spite of this, as a function of PT, performance for each choice type was essentially the same in the two conditions (Fig. 2a).

Using such split-data analysis, we also examined the degree to which perceptual performance was sensitive to other task-specific features. Similar results were obtained when the data were conditioned on the orientation of the targets (horizontal versus vertical), on the peak velocity of the choice saccades (high versus low), or on the total angular distance traversed by the eyes in each choice (short versus long; Fig. 2b). Qualitatively, the pro and anti tachometric curves always bore the same relation to PT and to each other regardless of how the data were partitioned. Quantitatively, these and other analyses yielded some differences across data subsets, but they were slight, indicating that oculomotor parameters relate only weakly to perceptual performance, as expected (Seideman et al., 2018).

Overall, the results so far show that, in spite of the relaxed task control and highly dynamic conditions, performance in SpotChase is robust and eminently consistent with that observed during trial-based urgent tasks: in both cases perceptual accuracy is fundamentally determined by the PT, the salience of the cue onset leads to reliable oculomotor capture early on, and this exogenous (involuntary) draw can be overcome slightly later by an endogenous (voluntary) signal that is congruent with the task goals.

### Early non-random responses are salience-driven

Prior studies with urgent tasks indicate that the pronounced dip below chance observed during blue-cue choices is due to involuntary, exogenous capture that is driven by stimulus salience and, for the most part, acts independently of endogenous goals or task rules (Goldstein et al., 2022; Oor et al., 2023). To determine whether this was indeed the case under dynamic conditions, 9 of our 19 participants performed the salient-noncue (**SNC**) variant of SpotChase (Methods).

**Figure 3.**
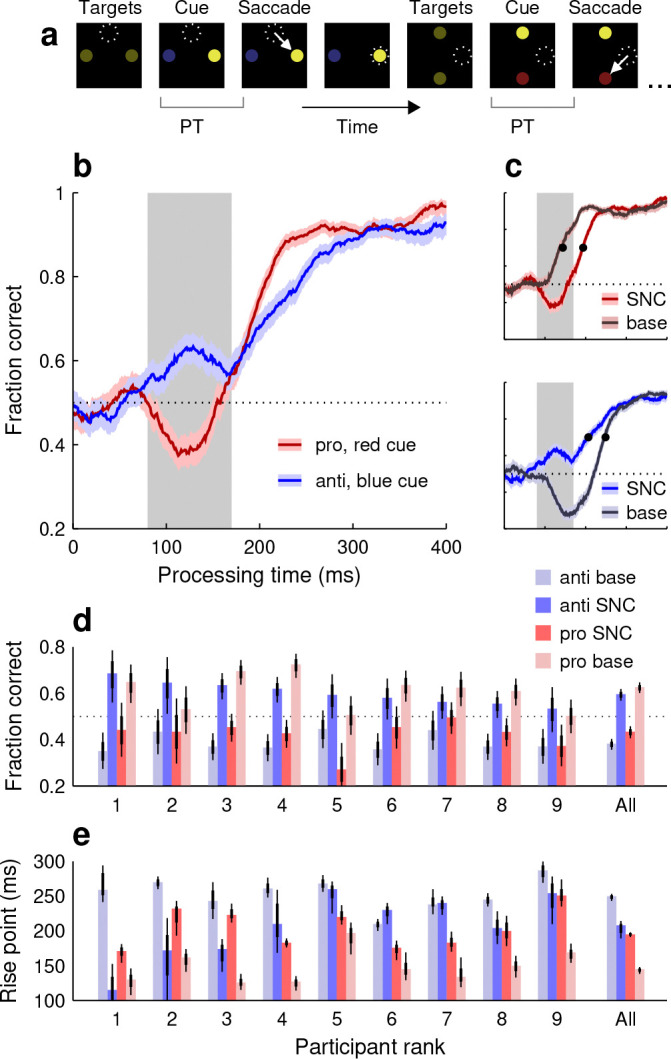
Performance in the salient-noncue (SNC) variant of SpotChase. **a**, Sequence of events in the task. The initial yellow targets are of low luminance. Following a variable time, one target (cue) turns red or blue and remains of low luminance, whereas the other (noncue) stays yellow but shifts to high luminance. The task is otherwise the same as in the base version. **b**, Tachometric curves for red-cue, or pro choices (red trace) and blue-cue, or anti choices (blue trace) pooled across 9 participants. Shaded ribbons indicate 95% CIs across choices. Shaded rectangle marks the exogenous capture window used for analysis (80 ≤ PT ≤ 170 ms). Horizontal dotted line indicates chance level. **c**, Comparison between tachometric curves in the SNC (bright colors) and base (dark colors) variants. Same conventions as in **b**. Upper and lower graphs show pro (red traces) and anti curves (blue traces), respectively. Data for all curves are from the same participants. Black dots mark curve rise points. **d**, Fraction of correct responses within the exogenous capture window for individual participants. Bars indicate results for pro (red) and anti (blue) choices in the SNC (saturated colors) and base conditions (light colors). Thin and thick lines mark 95% and 68% CIs, respectively. Participants are sorted by their effect magnitude in the anti SNC case. **e**, Curve rise points across participants. Same conventions and participant order as in **d**.

The SNC variant followed the same layout as the base, but the luminances of the stimuli were different (Fig. 3a). Recall that, in the base version, the cue was more salient than the opposing target (noncue) because of its sudden color change. In the SNC version, the initial yellow targets were of low luminance. Then, after a variable time period, one target (cue) switched to a low-luminance red or blue while the other (noncue) turned to a high-luminance yellow. The idea was for the noncue, which was still behaviorally uninformative, to become more salient than the cue, which was still behaviorally informative.

As before, separate tachometric curves were generated for pro (red-cue) and anti (blue-cue) choices (Fig 3b). Now, the early deviations from chance (PT ≳ 80 ms) were reversed relative to the trends seen in the base condition. During anti choices (Fig. 3b, blue trace), performance showed an initial, transient uptick above chance consistent with exogenous oculomotor capture toward the salient noncue, i.e., toward the correct response. Conversely, during pro choices (Fig. 3b, red trace), performance showed precisely the opposite, a brief dip. This downward deflection was again consistent with exogenous capture toward the noncue, but in this case it corresponded to the incorrect response.

To quantify this exogenous pull, for each participant we computed the fraction correct based on all the pro or anti choices found within a restricted exogenous capture window (80 ≤ PT ≤ 170 ms; Fig. 3b, c, gray shades; Methods). Results were highly uniform across our sample (Fig. 3d): whereas in the base task performance in this window was consistently higher for pro than for anti choices (all light red bars higher than light blue bars), in the SNC variant the opposite was true, performance was consistently higher for the anti choices (all bright blue bars higher than bright red bars). Reversing the direction of salience reliably reversed the early deflections in performance.

Additionally, the SNC results showed that the timing of the later, endogenously driven rise toward asymptotic accuracy was also impacted by the exogenous signal. Whether the correct choice was toward the cue (Fig. 3c, top graph) or toward the noncue (Fig. 3c, bottom graph), an exogenous pull away from the correct choice tended to delay the endogenously driven rise. For pro choices, the rise point for the pooled data increased by 50 ms as the salience signal shifted from the cue to the noncue (rise point in base version: 144 ms in [138, 148] ms, 95% CI; rise point in SNC version: 194 ms in [191, 198] ms, 95% CI; Fig. 3c, top, black dots). And for anti choices, the rise point increased by 42 ms as the salience signal shifted from the noncue to the cue (rise point in SNC version: 207 ms in [189, 214] ms, 95% CI; rise point in base version: 249 ms in [243, 253] ms, 95% CI; Fig. 3c, bottom, black dots). These delays were also highly consistent across individual participants (Fig. 3e; note all bright red bars above light red bars, and light blue bars above bright blue bars in 7 of 9 cases). When the salience signal was misaligned with the goal, it took longer to elaborate a correct informed choice.

These characteristic effects correspond to the simultaneous occurrence of two phenomena that have been typically considered separately. The early deviations from chance performance represent involuntary saccades toward a salient stimulus, and are akin to traditional oculomotor capture (Theeuwes et al., 1998, 1999). In turn, the delays in reaching a performance criterion represent a time cost on voluntary saccades toward a goal, and are akin to traditional attentional capture (Ruz and Lupiáñez, 2002; Theeuwes, 2010; Luck et al., 2021). These overt and covert forms of exogenous capture, which may be understood as distinct manifestations of the same underlying mechanism (Salinas et al., 2019; Salinas and Stanford, 2021; Goldstein et al., 2022; Oor et al., 2023), occur together and quite robustly under dynamic, less constrained viewing conditions.

### Advanced knowledge of cue location engages endogenous attention

One of the most characteristic aspects of visuospatial attention is that it can be deployed voluntarily and covertly, independently of gaze (Eckstein et al., 2004; Ignashchenkova et al., 2004; Thompson et al., 2005; Busse et al., 2008; Zhou and Thompson, 2009; Herrington and Assad, 2010; Carrasco, 2011). This has been amply demonstrated in tasks that require sustained fixation. However, other evidence suggests that the voluntary deployment of attention is generally accompanied by an incipient motor plan to the attended point (Kustov and Robinson, 1996; Belopolsky and Theeuwes, 2009; Hanning et al., 2022; Goldstein et al., 2024). To investigate the coupling between endogenous, covert attention and saccadic choices under less constrained conditions, we designed a variant of our dynamic task in which participants could potentially benefit from deploying attention to specific locations in advance of their eye movements.

**Figure 4.**
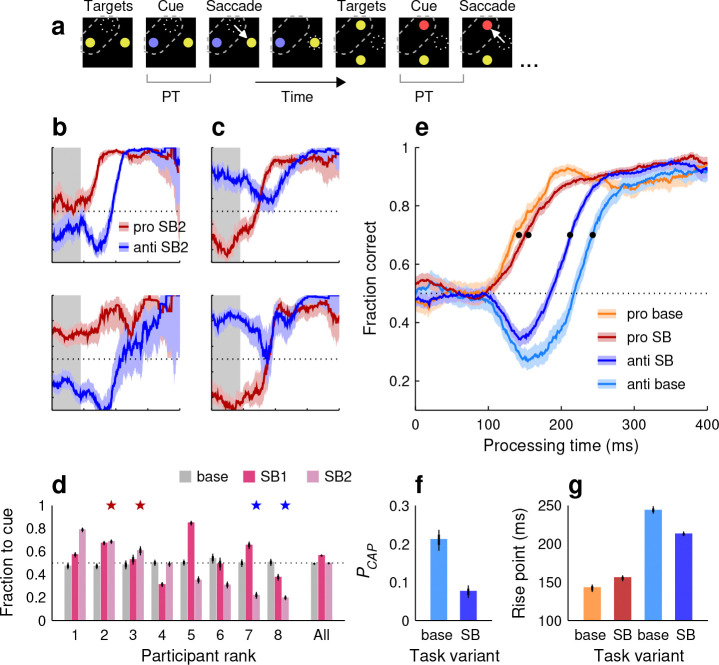
Performance in the spatial-bias (SB) variant of SpotChase. **a**, Sequence of events in the task. Participants knew that the informative red or blue cues would always appear at fixed locations, top or left in this example (SB1; dashed outlines, not shown on screen). Otherwise, the task proceeded as in the base version. **b**, Tachometric curves from two participants who demonstrated a bias toward the known cue location. See panel **e** for axis labels. Shaded ribbons indicate 95% CIs across choices. Shaded rectangles mark the response-bias window (PT ≤ 90 ms). **c**, As in **b**, but for two participants whose responses were biased away from the known cue location. **d**, Fraction of uninformed choices (guesses) toward the cue location for each participant; PT window used is indicated in **b**, **c**. Bars depict results when the cue appeared unpredictably (base, gray), when it appeared predictably at the top or left locations (SB1, dark pink), and when it appeared predictably at the bottom or right locations (SB2, light pink). Participants are sorted by their effect magnitude in the SB2 case. Stars mark the example participants in **b** (red pair) and **c** (blue pair). **e**, Comparison between pooled tachometric curves in the SB (dark colors; SB1 and SB2 data combined) and base (light colors) task variants from the same participants. Black dots mark curve rise points. **f**, Probability of capture for the anti curves shown in **e**. **g**, Rise points for the four curves shown in **e**. In **d**, **f**, **g**, thin and thick lines mark 95% and 68% CIs, respectively.

In the spatial bias (**SB**) variant of SpotChase (Fig. 4a; Methods), participants were made aware, before the start of each run, that the red or blue cue would always appear either at the top and left positions (SB1 runs), or at the bottom and right positions (SB2 runs). Participants were not instructed to perform any differently; they were simply informed of the spatial regularity. This way, they had the opportunity to covertly attend to the informative cue locations early on — but note that this did not give away what the correct choice was, because the cue color (red or blue) remained unpredictable. The spatial regularity could bolster performance only in that it could potentially save some processing time by expediting the identification of the cue color. In fact, initially we contemplated the possibility that performance in this variant of the task might be the same as in the base, because all the colored stimuli were of high luminance and easily discriminable from each other to begin with.

Nevertheless, the participants’ behavior did change quite noticeably. During uninformed choices, some participants tended to make more saccades to the cue (Fig. 4b), whereas others tended to make more in the opposite direction, to the noncue (Fig. 4c). We calculated the mean fraction of choices toward the cue in the guessing range of processing times (PT ≤ 90 ms). By this measure, guesses in the SB variant were typically biased for any given participant (Fig. 4d; fraction to cue was significantly different from 0.5 with $p<10^{-6}$ in 13 of 16 cases), much more so than in the base condition (Fig. 4d; mean absolute deviation from 0.5 in base condition: 0.015; in SB condition: 0.157; p=0.0012, permutation test). On average, though, the bias in the pooled data was very small (Fig. 4d, All; Fig. 4e, bright red and blue traces). The varied directions and magnitudes of the observed biases, suggests that participants indeed attended to the cue locations, sometimes looking directly at the salient cue and sometimes attempting not to do so (Goldstein et al., 2024), as if searching for an ideal strategy. In any case, although it manifested in different trends across participants, knowledge of the cue location certainly had an impact on their viewing behavior.

But, was knowing the location of the cue ultimately beneficial to performance? The evidence suggests that yes, on average, covertly attending to the cue locations did bolster perceptual processing specifically during anti choices (Fig. 4e, blue curves). In comparing the pooled data from the same participants during the base and SB versions, we found that the anti tachometric curve rose about 31 ms earlier when the cue location was known (Fig. 4g, blue bars), with the fraction of captured saccades decreasing substantially also (Fig. 4f). Variations during pro choices were much smaller in comparison (Fig. 4e, g, red traces and bars). The observed changes in perceptual processing (i.e., via the anti tachometric curve) were consistent with improved run scores, which were significantly higher during SB than during base runs ($p<10^{-6}$, permutation test). This suggests that the speeded processing of the blue cues did translate into higher success relative to the stated goal of the task.

**Figure 5.**
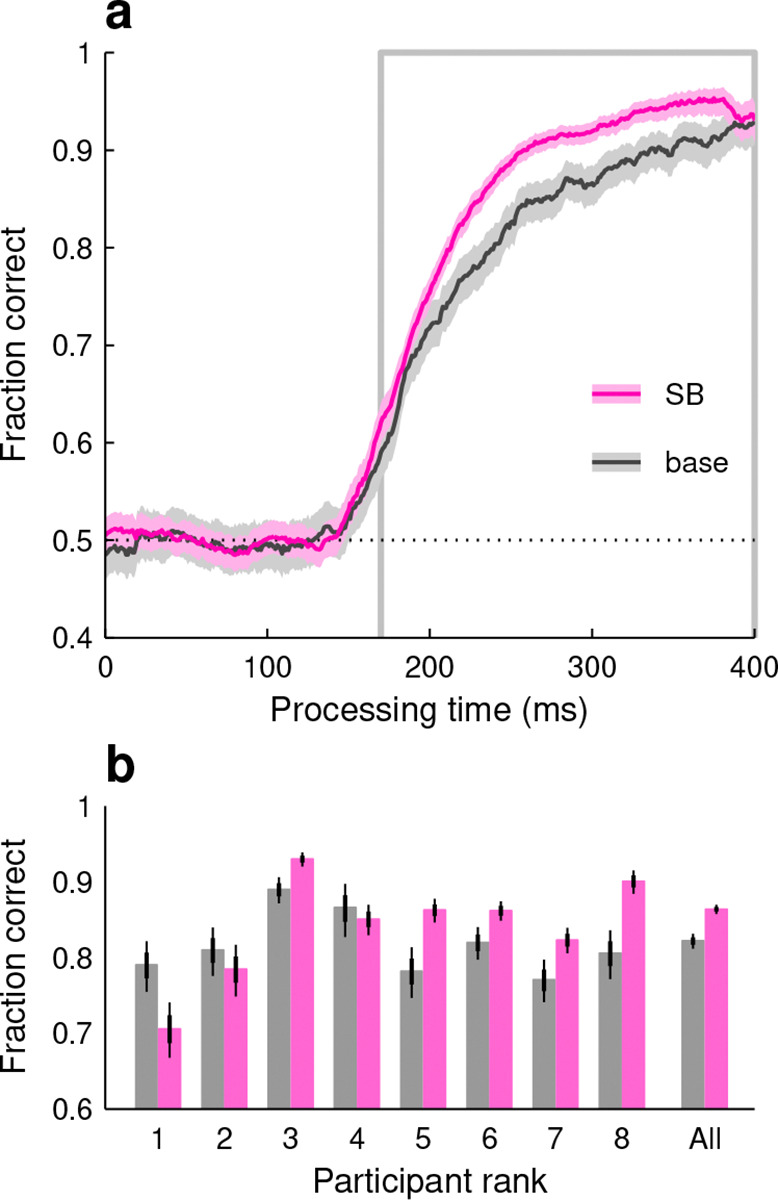
Advanced knowledge of the cue location tended to improve performance. All data are from a group of 8 participants who performed the base and spatial-bias (SB) variants of SpotChase. All results are for pro (red-cue) and anti (blue-cue) choices combined. **a**, Pooled tachometric curves for the SB (magenta) and base (gray) variants. Shaded ribbons denote 95% CIs. Outlined gray rectangle marks the PT window used for analysis (170 ≤ PT ≤ 400 ms). **b**, Fraction of correct responses within the analysis window (indicated in **a**). Results for individual participants in the base (gray) and SB (magenta) variants. Thin and thick lines mark 95% and 68% CIs, respectively. Same participant order as in Fig. 4d.

In this case, the measurement of $P_{CAP}$ and rise point from single-subject curves was hindered by the participants’ strong biases. So, instead, we considered the performance of individual participants in pro and anti choices combined. This way, the biases cancelled out, yet the higher perceptual efficiency during the SB variant remained evident (Fig. 5a). With this approach, we confirmed that the effect observed in the pooled data (Fig. 4e–g) was largely consistent across individuals: the combined fraction correct in the SB condition was clearly higher in 5 of 8 participants, similar in 2 of them, and clearly lower in only 1, judging by the overlap between CIs (Fig. 5b).

In summary, we observed perceptual improvement that was as expected from the voluntary allocation of attention to the cue locations and generally consistent across our sample. The widely varying motor biases exhibited by the participants suggests that the spatial correlation between attentional deployment and oculomotor planning is weak even without strict fixation.

### Perceptual performance is strongly swayed by stimulus statistics

Expectations and statistical regularities in the environment can exert profound influence on perception and attention (Sterzer et al., 2008; Seriès and Seitz, 2013; de Lange et al., 2018; Rungratsameetaweemana and Serences, 2019; Klink et a., 2023). To assess the effects of stimulus (red/blue cue) or task (pro/anti) statistics on visuomotor performance, ten participants completed a new variant of SpotChase, one with variable pro-anti ratio (**PAR**; Methods). In the PAR version, red and blue cues were still presented randomly but one color was sampled more frequently than the other. The ratio of pro-to-anti (or red:blue) choices was 3:1, 6:1, 1:3, or 1:6, and each ratio yielded a full dataset from each of the ten participants. We generally expected that performance would be somewhat better for the overrepresented choice type than for the underrepresented; however, given an earlier study where only minimal differences were observed between blocked and interleaved pro/anti trials (Goldstein et al., 2022), strong effects were not anticipated.

**Figure 6.**
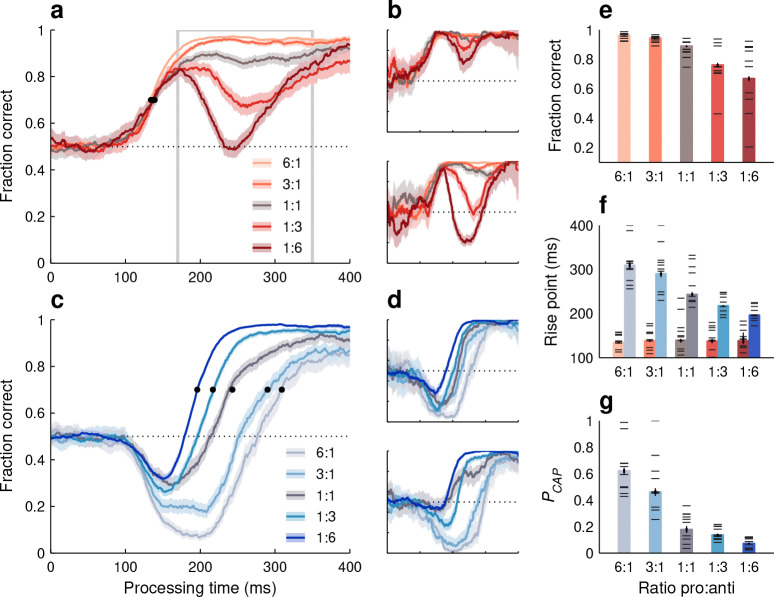
Performance in the variable pro-anti ratio (PAR) variant of SpotChase. **a**, Tachometric curves for red-cue, or pro choices pooled across participants. Each curve corresponds to a different pro:anti ratio, as indicated. Shaded ribbons indicate 95% CIs across trials. Outlined gray rectangle marks the endogenous-response window (170 ≤ PT ≤ 350 ms). Horizontal dotted line indicates chance level. Black dots mark curve rise points. All curves are from the same 10 participants. **b**, As in **a**, but for two individual participants. **c**, Tachometric curves for blue-cue, or anti choices pooled across participants. Same conventions as in **a**. **d**, As in **c**, but for two individual participants. **e**, Fraction of correct pro choices in the endogenous response window (outlined in **a**) for each pro:anti ratio. **f**, Rise points for the pro (red bars) and anti (blue bars) tachometric curves for each pro:anti ratio. **g**, Probability of capture during anti choices for each pro:anti ratio. In **e**–**g**, data from individual participants (horizontal lines) are superimposed on results from pooled curves (bars), and thin and thick vertical lines mark 95% and 68% CIs for the pooled results. All 1:1 data (gray traces and bars) are from the base version of the task.

Actually, the impact of stimulus/task statistics was surprisingly large, both for red-cue (Fig. 6a, b) and blue-cue choices (Fig. 6c, d). In general, relative to the base condition (1:1 ratio), performance improved substantially when a given choice type occurred at a high frequency and deteriorated substantially when it occurred at a low frequency, but unique trends were observed for each cue color. During red-cue choices, the early rise in performance was unaffected by the task statistics, so the rise point of the pooled tachometric curves did not change (Fig. 6a, black dots; Fig. 6f, red bars). However, at later PTs (170 ≤ PT ≤ 350 ms), when choices are informed by the task rules, the fraction correct reached 0.96 as red-cue choices became more common and fell to around 0.67 as they became more rare (Fig. 6e). In contrast, during anti choices, the pooled tachometric curves shifted markedly according to the frequency of the blue cues (Fig. 6c, black dots; Fig. 6f, blue bars), with the rise point going as low as 196 ms (in [195, 198] ms, 95% CI) for the 1:6 ratio and as high as 309 ms (in [301, 319] ms, 95% CI) for the 6:1 ratio. The task statistics determined not only the timing of the rise in anti performance, but also the proportion of captured saccades, which varied dramatically as well (Fig. 6g).

These findings were generally consistent for individual participants, with an interesting pattern visible in all the quantitative measures: the variability across subjects was always much smaller when the mean performance was high than when it was low. For instance, during endogenously guided pro choices (Fig. 6e, horizontal lines), all participants achieved a fraction correct above 0.92 with the 6:1 ratio, whereas with the 1:6 ratio, the fraction correct varied widely (range: [0.2, 0.92]) around a lower mean (of 0.68). A similar distinction was observed during anti choices, both for $P_{CAP}$ (Fig. 6g, horizontal lines) and for the curve rise point (Fig. 6f, horizontal lines on blue bars). When a task (pro or anti) was encountered often, perceptual performance was uniformly accurate; when the same task was infrequent, not only was performance poorer on average, but also the amount of deterioration varied quite broadly across participants. Individual differences are analyzed in more detail below.

### Selection history accounts for sensitivity to task statistics

**Figure 7.**
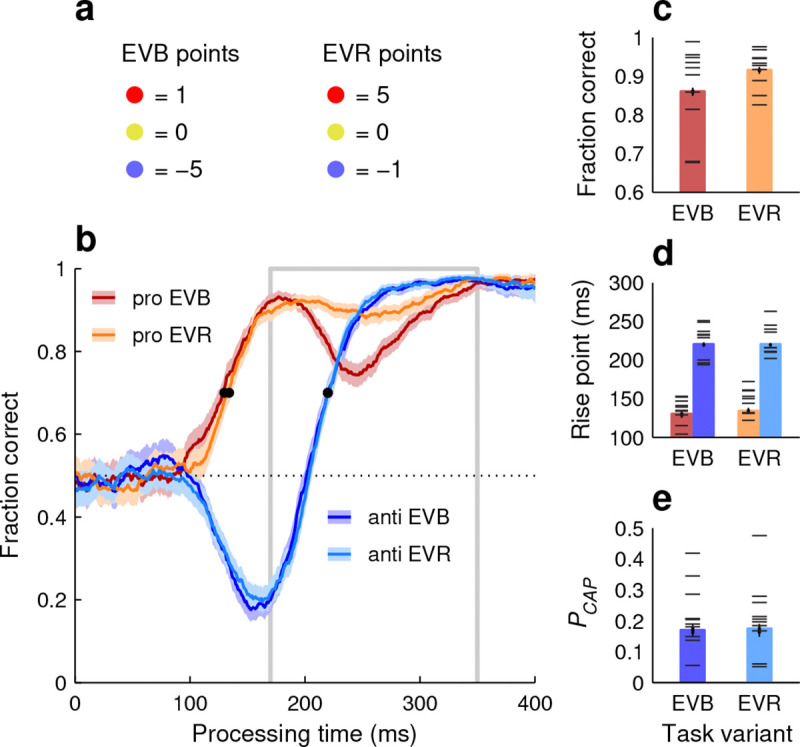
Performance in the extreme-value (EV) version of SpotChase. **a**, Point values assigned to each cue color in the EVB (anti choices prioritized) and EVR (pro choices prioritized) task variants. **b**, Pooled tachometric curves for pro (red) and anti (blue) choices in the EVB (dark colors) and EVR (light colors) variants. Shaded ribbons indicate 95% CIs across choices. Outlined gray rectangle marks the endogenous-response window (170 ≤ PT ≤ 350 ms). Black dots mark curve rise points. **c**, Fraction of correct pro choices in the endogenous response window (outlined in **b**). **d**, Rise points for pro (red) and anti (blue) tachometric curves. **e**, Probability of capture during anti choices. In **c**–**e**, data from individual participants (horizontal lines) are superimposed on results from pooled curves (bars); thin and thick vertical lines mark 95% and 68% CIs, respectively. All data are from the 10 participants who performed the PAR and EV variants.

We considered two mechanisms potentially responsible for the observed sensitivity to task statistics. The first was top-down modulation. In this scenario, performance varies between pro-anti ratios because participants recognize a dominant choice type and strategically prioritize it at the expense of the alternative type. To test whether participants could voluntarily trade accuracy in one task against the other in such a way, the same 10 subjects who completed the PAR variant went on to perform the extreme-value (**EV**) variant of SpotChase. In this variant, everything was identical to the base version, including the 1:1 red-blue ratio, except that the point values assigned to each colored choice were different (Fig. 7a; Methods). In one case (EVB runs) blue-cue errors were much more costly than red-cue errors, whereas in another case (EVR runs) the reverse was true.

Participants responded to the different incentives as intended; specifically, their overall success rate for anti choices was higher during EVB runs (68.7% correct in [67.8, 70.0], 95% CI, pooled data) than during EVR runs (65.1% in [64.2, 66.1], 95% CI), whereas their overall success rate for pro choices did not change (74.6% in both cases). This, however, largely resulted from adjustments in urgency (pace); in terms of perceptual processing capacity, as quantified by the tachometric curve, the two priority settings yielded only minimal differences (Fig. 7). The pooled anti curves were essentially identical between EVB and EVR conditions (Fig. 7b, d, e, blue traces and bars), and comparisons based on individual participant data yielded no significant differences in $P_{CAP}$ or rise point (p>0.6 in both cases, permutation test; Fig. 7d, e, horizontal lines superimposed on blue bars). For the pooled pro curves, the early rise in performance was also the same across conditions (Fig. 7b, d, red/orange traces and bars). The only notable effect was a drop in the fraction correct in the later endogenous range (170 ≤ PT ≤ 350 ms) when blue-cue choices were prioritized (EVB case). This moderate decrease in performance was evident for the pooled data and clear (no overlap between 95% CIs) for 5 of the 10 participants (Fig. 7c).

Thus, in terms of perceptual processing, the exerted top-down modulation was ineffective, if not altogether counterproductive: the prioritization of anti choices (EVB case) did not improve anti performance at all relative to the opposite criterion (EVR case), it simply made the evaluation of pro choices slightly worse. This indicates that the strong modulation due to stimulus/task statistics was fundamentally involuntary.

With this in mind, we considered a second possible mechanistic explanation: selection history effects. To assess the influence of prior choices on task performance, the data from the PAR variant were split by choice type (pro or anti; or equivalently, red or blue cues) and sorted based on the history of cue colors that preceded each choice (Methods). We focused on the effect that 1, 2,… and up to 7 repetitions of a given cue color had on a subsequent choice of the same (S) or different (D) type. We refer to the resulting sequences as 7S, 6S,…1S, 1D, 2D…, 7D, where each case applies to red or blue cues. For example, 4S pro choices are red-cue choices that were preceded by four other consecutive same-color (red) choices; similarly, 2D anti choices are blue-cue choices that were preceded by two consecutive different-color (red) choices, etc. Finally, a history-conditioned tachometric curve was constructed for each of the sequences considered (28 in total).

**Figure 8.**
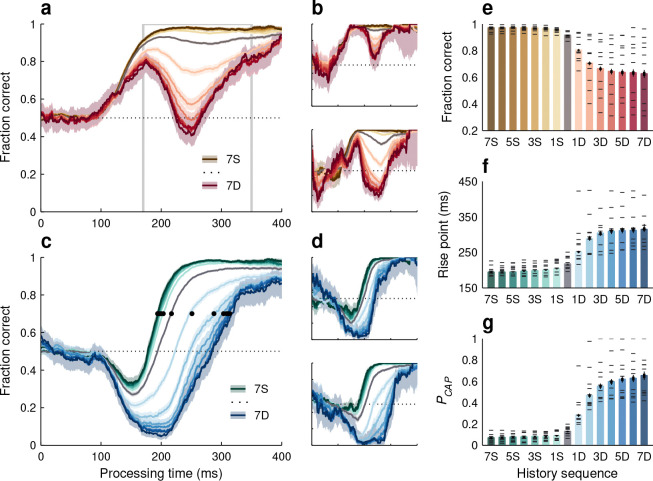
Perceptual performance depends strongly on recent history. Data are from the PAR variant but sorted by color history. For each choice type (pro or anti), data subsets were created where each choice was preceded by a series of N repeats of the same (S) or different (D) cue color. Same conventions as in Fig. 6. **a**, Tachometric curves for red-cue, or pro choices conditioned on preceding history and pooled across participants. Each curve is conditioned on a different color sequence going from 7 red-cue repeats (7S, dark brown) to 7 blue-cue repeats (7D, dark red) before a pro choice. Lighter colors indicate conditions with fewer repeats. The gray curve corresponds to average pro performance irrespective of history. **b**, As in **a**, but for two individual participants. **c**, As in **a**, but for for blue-cue, or anti choices, and with color sequences going from 7 blue-cue repeats (7S, dark green) to 7 red-cue repeats (7D, dark blue) before an anti choice. **d**, As in **c**, but for two individual participants. **e**–**g**, Behavioral metrics as functions of history sequence. Results are for the fraction of correct pro choices in the endogenous response window (**e**), and for the rise point (**f**) and probability of capture (**f**) for the anti tachometric curves. Gray bars in the middle correspond to average results irrespective of history.

We found that history had a profound impact on perception (Fig. 8). In general, performance improved when a given cue color was repeated from the previous choices to the current one, but deteriorated when a switch occurred. For red-cue or pro choices (Fig. 8a, b, e), large variations in success rate occurred during the endogenous response window (170 ≤ PT ≤ 350 ms), with the fraction correct in it going from 0.96 when a red-cue choice was preceded by seven red-cue choices to 0.63 when it was preceded by seven blue-cue choices (7S to 7D, pooled data). For blue-cue or anti choices, the relative shifts in the pooled tachometric curves spanned 120 ms (Fig. 8c, black dots; Fig. 8f, bars), with the rise point going between 194 ms (in [192, 197] ms, 95% CI) at the 7S end to 314 ms (in [307, 330] ms, 95% CI) at the 7D end. Color history yielded similarly vast differences in the proportion of captured saccades (Fig. 8g, bars). These effects were highly robust in both the pooled (Fig. 8a, c) and individual-participant data (Fig. 8b, d; see below).

**Figure 9.**
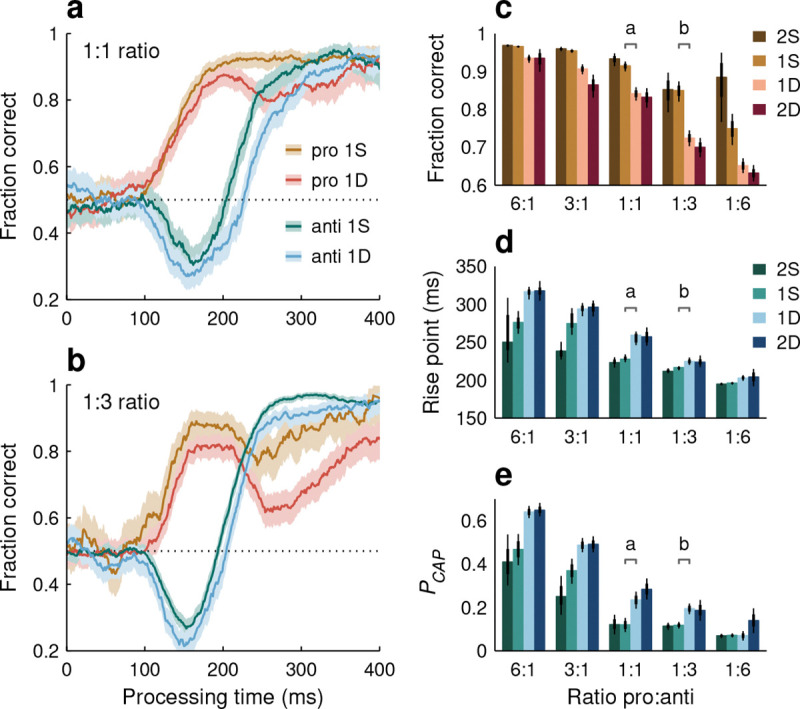
SpotChase performance depends on both short- and long-term history. For each pro:anti ratio dataset, data were pooled over participants, and pro and anti choices were conditioned on the prior history of cue colors (sequences 2S, 1S, 1D, 2D). **a**, History-conditioned tachometric curves from the 1:1 ratio (base) dataset. Curves for pro (brown, red) and anti (green, blue) choices are shown, each given a repeat (1S) or a switch (1D) in cue color relative to the preceding choice. Other conventions as in Fig. 6a. **b**, As in **a**, but for data from the 1:3 ratio. **c**–**e**, Behavioral metrics as functions of pro:anti ratio and history sequence. Bars indicate the fraction of correct pro choices in the endogenous response window (**c**), and the rise point (**d**) and probability of capture (**e**) of the anti tachometric curve. Results corresponding to the tachometric curves in **a** and **b** are indicated.

The qualitative similarity in performance between different ratio conditions (Fig. 6) and different history sequences (Fig. 8) is apparent and not entirely surprising, because the history analysis just presented was based on all the ratio conditions combined; thus, it provided a more finely stratified breakdown of the variance found in the PAR data. Another way to appreciate the impact of selection history, and the different timescales responsible for its full effect, is to parse the data according to history but doing so separately for each ratio condition. This way, the variance in performance due to recent, local fluctuations in cue color can be distinguished from the variance due to more distant, global fluctuations (Fig. 9).

When the data from individual ratio conditions were sorted by recent history going back one or two choices, we confirmed that selection history effects were always present and that perceptual performance was generally better when the cue color was repeated (Fig. 9, S sequences) than when it switched (D sequences). Qualitatively, variations between repeats and switches were similar across ratio conditions, as can be seen by comparing, for instance, the tachometric curves for the 1:1 (Fig. 9a) and 1:3 (Fig. 9b) datasets. Quantitatively, the three metrics used to characterize performance demonstrated the same three patterns. (1) Most often, the 1S condition yielded a substantially better result than the 1D condition (Fig. 9c–e, no overlap in 95% CIs for 1S versus 1D bars for 13 of 15 cases). Compared to a switch, one color repetition consistently led to a higher fraction correct in pro choices and a lower $P_{CAP}$ and rise point in anti choices. (2) The results were generally similar for one versus two repetitions of a given color (Fig. 9c–e, overlap in 95% CIs in 14 of 15 cases for both 2S versus 1S bars and 2D versus 1D bars). Taking into account two trials into the past was only slightly more predictive than taking into account only one (Fig. 9c–e, differences between 2S and 2D values tended to be larger than differences between 1S and 1D values; p>0.06, permutation tests). (3) In all three metrics, there was substantial variation both across histories and across ratios (p<0.002 for history, $p<2\times 10^{-5}$ for ratio, two-way ANOVAs).

We interpret these patterns as follows. The consistent influence of immediate history (1S, 1D contrast) reveals the action of some adaptation mechanisms that are fast and local. However, their apparent saturation (2S, 1S and 2D, 1D contrasts) does not imply the absence of other, slower mechanisms that produce cumulative effects (ratio contrasts). These may go largely undetected unless the analyses can go farther into the past. Thus, the full-blown impact of past events becomes evident only when the task statistics are manipulated to permit reliable comparisons across longer histories (e.g., 7S, 7D contrast).

The history of prior cue colors produced large variations in performance in SpotChase. Now, given that reward (or success) is a potent driver of behavior and modulator of attention (Milstein and Dorris, 2007; Chelazzi et al., 2013; Failing and Theeuwes, 2018; Oor et al., 2024), we also asked: was the history of prior outcomes (correct or incorrect) also relevant to such variations? The answer is essentially “no.” The PAR data were again split by choice type (pro or anti), but this time they were sorted according to outcome history. We considered all the choices of a given type that were preceded by one correct choice (1C), two correct choices (2C), etc., or by one error (1E), two errors (2E), etc. The resulting tachometric curves conditioned on outcome history largely overlapped each other, and the three metrics used for characterizing them demonstrated minimal variations across sequences. Analyses combining both outcome and cue-color histories did not reveal a substantial effect of outcome either. Thus, the trial-by-trial history effects were fundamentally feature-based.

### Selection history effects characterize individual variability

Earlier we noted that, when the task was relatively difficult, performance varied widely between participants (Figs. 6e–g, 8e–g, black horizontal lines). This was true for both pro and anti choices, and we initially thought that the two effects would be consistent; that is, that each participant would demonstrate a particular sensitivity to history regardless of choice type. Interestingly, however, the history dependencies for pro and anti choices turned out to be unrelated.

**Figure 10.**
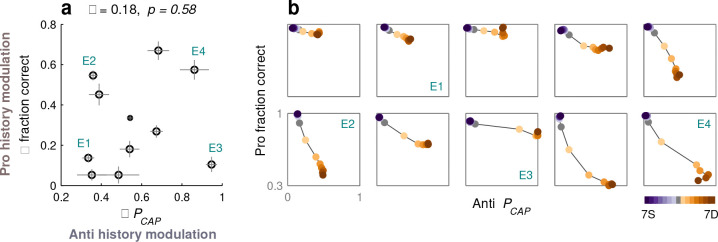
Selection history effects characterize individual variability. **a**, Sensitivity to cue-color history in pro choices (Δ fraction correct) versus in anti choices ($\Delta P_{CAP}$). Each circle corresponds to one participant. Error bars indicate ± 1 SE. For reference, the small point shows results for the pooled data. Pearson correlation coefficient and significance (from permutation test) are indicated. **b**, Joint, history-driven variations in pro and anti performance. Each square contains data from one participant, and shows the fraction correct in pro choices (y axis) versus the probability of capture ($P_{CAP}$) in anti choices (x axis) across history sequences (colored dots; see colorbar). Same data as in Fig. 8e, g, but sorted by participant. Labels correspond to the four example participants marked in **a**.

For each participant who performed the PAR variant of SpotChase, two quantities were calculated to describe their sensitivity to cue-color history, a Δ fraction correct (during pro choices) and a $\Delta P_{CAP}$ (during anti choices). Each Δ was equal to the (positive) difference between the 5S and 5D values of the corresponding behavioral metric derived from the history analysis (Fig. 8e, g; results were similar for sequences with ≥ 3 repetitions). The pairing of these two quantities produced data points that were widely spread (Fig. 10a). For some participants, both values were indeed small (e.g., E1) or large (e.g., E4), indicating similarly weak or similarly strong sensitivity to history during pro and anti choices alike. But overall there was no correlation; for other participants (e.g., E2, E3), the modulation was large in one direction but small in the other.

The range of individual sensitivities becomes even more evident when the fraction correct from pro choices and $P_{CAP}$ from anti choices are plotted against each other to visualize how they change together as a function of history sequence (Fig. 10b). In this representation, the data may stay near the top left corner (highest accuracy in pro and anti choices; E1), may transition primarily toward the right (E3) or bottom edge (E2), or may end near the bottom right corner (lowest accuracy in pro and anti choices; E4). These results are notable because they indicate not only that sensitivity to recent history is a major source of individual variability in perceptual capacity, but also that there are multiple, seemingly equivalent but distinct adaptation mechanisms that make independent contributions to such variability.

## Discussion

SpotChase produces viewing conditions that are more dynamic and less constrained than in traditional, trial-based tasks, yet it permits rigorous quantification of perceptual capacity. This revealed clearly identifiable contributions to visuomotor performance from multiple attention mechanisms within a continuously flowing behavior.

Studying cognition in more naturalistic settings is critical because even highly established phenomena can change unpredictably when stimulation becomes more realistic and behavioral control less stringent (Camerer and Mobbs, 2017; Snow and Culham, 2021). For instance, an experiment using gaze-contingent rendering in an immersive virtual reality display demonstrated a remarkable degree of change blindness (Cohen et al., 2020). While observers explored color visual environments, the periphery beyond the central 10–30° gradually turned black-and-white, but observers were often unaware of the blatant manipulation. Voluntarily attending to the periphery (or not) was highly consequential in this case. Here, the most unexpected result was that history-driven mechanisms exerted an involuntary, surprisingly powerful influence on perceptual performance. We also found that the interplay between stimulus-driven and goal-driven mechanisms was qualitatively similar to that observed in urgent, trial-based tasks — but the corresponding data were also interesting. For instance, they showed that there are benefits to the covert allocation of attention even when visual scenes consist of highly discriminable stimuli, change rapidly, and yield idiosyncratic saccade planning (Fig. 7).

To appreciate the results, it is worth stressing the fundamental distinction between the RT, which is anchored to the start of motor planning (here, onset of targets), and the PT, which is anchored to the start of perceptual processing (here, cue onset). Whereas the RT is a direct reflection of urgency, i.e., how rapidly motor plans advance, the PT is not. Thus, the behavioral curve that describes choice accuracy as a function of PT, the tachometric curve, quantifies performance independently (mostly) of motor urgency (Fig. 2a). Furthermore, it can be read as a representation of how the probability of success changes over time *during a single choice* (Salinas et al., 2010; Stanford et al., 2010; Costello et al., 2013; Seideman et al., 2018; Salinas et al., 2019; Oor et al., 2023).

With this in mind, the evolution of the covert selection process during a choice consisted of four distinct phases.

1. Motor plans are launched early on but are initially uninformed. Any saccades produced with PT . 100 are either random guesses (Fig. 1b–d) or internally biased guesses (Fig. 4b–d).
2. The initial impact of the cue becomes evident after the 100 ms mark, when accuracy departs systematically from chance (Fig. 1b–d). The underlying signal driving this departure has all the hallmarks of an involuntary bias: it is fast, it tracks the most salient stimulus regardless of task rules (Fig. 3b), and its effects are uniform across individuals (Fig. 3d, e). It can be thought of as a transient (100 ≲ PT ≲ 170 ms), stimulus-driven pulse of neural activity that automatically biases ongoing motor plans toward salient, recently detected targets (Bisley et al., 2004; Ipata et al., 2006; Scerra et al., 2019; Goldstein et al., 2022; Klink et al., 2023; Zhu et al., 2024).
3. Following the early exogenous pulse, motor plans are steered toward the correct choice by a later endogenous signal that also depends on the cue, but after it has been interpreted according to task rules. This signal arrives about 150 ms after cue onset, but its timing can change substantially depending on task conditions and individual participants (Fig. 3b, Figs. 6, 8).
4. Late in the target selection process (PT ≳ 350 ms), the exogenous pulse has faded and the endogenous signal has acted long enough for the correct motor alternative to prevail in most cases, be it toward or away from the cue (Fig. 1b–d; Fig. 3b, c; Fig. 4e; Fig. 7b). Errors at this point are akin to “lapses,” and may have a variety of interpretations (Pisupati et al., 2021; Ashwood et al., 2022; Gupta et al., 2024).

Thus, between 100 and 350 ms after cue onset, the dynamics of attention control and target selection are remarkably lawful and rich. Indeed, distinctive neurophysiological markers of attentional processing occur in this range (Luck, 2012). Resolving these dynamics psychophysically is key for relating behavior to neural activity. For instance, whereas the time point at which a neural circuit discriminates two spatial locations can be determined with high accuracy (~ tens of ms), the equivalent time point for the behavioral judgement generally has a much larger margin of error (Bisley and Goldberg, 2003; Busse et al., 2008; Cohen et al., 2009; Herrington and Assad, 2010). When the measurement is based on PT, however, the two can be directly compared at high resolution (Seideman et al., 2022).

During SpotChase, the abovementioned timecourse of events clearly describes how visuomotor performance is shaped by the spatial alignment and temporal ovelap between stimulus-driven and goal-driven signals.

When the exogenous pulse of activity is not aligned with the rule-based signal (anti choices), it produces either overtly captured, erroneous saccades or correct saccades with processing delays in comparison with the aligned condition (Figs. 1, 3) — effects analogous to traditional oculomotor and attentional capture (Theeuwes et al., 1998, 1999; Ruz and Lupiáñez, 2002; Luck et al., 2021; Theeuwes, 2010), as mentioned earlier. Such effects are not exclusively tied to physical salience. For instance, Poth (2021) reported qualitatively similar findings using urgent versions of classic cognitive tasks in which physical salience plays no role (a spatial Stroop task and a flanker task). We suspect that capture phenomena are the signatures of a more general and ubiquitous type of conflict, that between low-level (rapid, easily available) and high-level (slower, computationally expensive) signals (Kahneman, 2011; Aagten-Murphy and Bays, 2017; Poth, 2021). These signatures are fully revealed as functions of PT.

When the endogenous and exogenous signals are aligned (pro choices), they overlap and are difficult to dissociate (Fig. 1b–d, red traces; Aagten-Murphy and Bays, 2017; Goldstein et al., 2022). But the PAR and EV variants of SpotChase provided particularly clean evidence of their separate contributions: whereas the initial, stimulus-driven rise in accuracy changed very little, the later responses (PT ≳ 170 ms) varied dramatically with task statistics (Fig. 6a, b, e, f) or selection history (Fig. 8a, b, e), and to a lesser degree with top-down priority (Fig. 7b–d). The temporal and qualitative distinctions between exogenous and endogenous signals were stark. Normally, however, whenever an intended saccade target is salient, the informed, goal-driven signal seamlessly prolongs the effect of the earlier stimulus-driven pulse, so target selection appears to be guided by a single continous process.

The alignment of stimulus-driven and goal-driven signals was responsible for the patently different performance profiles of pro and anti choices, but the history of past events greatly amplified the differences (Figs. 6, 8). This is the most significant finding here. During anti choices, history modulated both the early, exogenous draw toward high salience and the later, endogenous draw toward high value (Fig. 8c, d, f, g). During pro choices, the same history instead seemed to modulate only the late, endogenous component (Fig. 8a, b, e). These findings are not necessarily inconsistent, because variations in particular behavioral metrics (here, $P_{CAP}$ and rise point) are not one-to-one correlates of variations in underlying neural activity. Specifically, during target selection, the goal-driven neural activity is thought to play two complementary roles: boosting the motor plan toward the correct choice, and curtailing the competing plan toward the incorrect one (Stanford et al., 2010; Costello et al., 2013; Salinas et al., 2019; Goldstein et al., 2022). The latter, suppressive component could directly impact the timing and likelihood of captured saccades (Salinas et al., 2019; Zhu et al., 2024). Thus, exogenous capture, as observed empirically, may partly depend on a particular component of endogenous control. In summary, while the impact of history on the goal-driven signal was definitely large, that on the stimulus-driven signal itself was probably weak.

The dynamic, uninterrupted viewing conditions of SpotChase likely created an ideal environment for selection history effects to manifest. These were complex, in that they (1) involved short and long time scales (Fig. 9), (2) varied widely across individual participants (Fig. 10), and (3) seemed to consist of separate, independent components associated with pro and anti choices (Figs. 8, 10). The findings are suggestive of task-switching costs (Wylie and Allport, 2000; Monsell, 2003; Stoet and Snyder, 2007), but with perceptual sensitivity instead of cognitive set. Relative to most prior studies documenting experience-driven biases on perceptual performance, the observed variations were huge (Figs. 6, 8), especially given that the stimuli were highly discriminable (for a detailed discussion of PT-dependenthistory effects compared to the wider literature, see Oor et al., 2024). The data, however, were in line with a recent study (Adam et al., 2024) demonstrating that a salient distracter presented for the very first time produces much stronger attentional capture than in typical experiments in which conditions are repeated. Expectation and/or recent experience matters, indeed.

The versatility and rigor of SpotChase should allow adaptation to clinical settings, where it has the potential to assess alterations in attention and cognition during disease states.

## Materials and Methods

### Subjects and setup

Experimental participants were 19 healthy human volunteers (13 females, 6 males; age range: 21–57 years) recruited from the Wake Forest Baptist Medical Center and broader Winston-Salem communities. All participants had normal or corrected-to-normal vision and provided written informed consent prior to the experiment. All experimental procedures were approved by the Institutional Review Board of Wake Forest University School of Medicine in accordance with protocol IRB 00035241.

Similar to a previous study (Goldstein et al., 2022), all experimental sessions were conducted in a dimly lit room. Participants were seated on an adjustable chair with their forehead and chin supported. Stimuli were presentedusing a VIEWPixx LED monitor (VPixx Technologies Inc., Saint Bruno, Quebec, Canada; 1920 × 1200 screen resolution, 120 Hz, refresh rate, 12-bit color) placed 57 cm in front of the participant. Eye position was monitored and recorded using an EyeLink 1000 infrared camera and tracker (SR Research, Ottawa, Canada; 1000 Hz sampling rate). All stimulus presentation and data collection routines were implemented using Matlab (Mathworks, Natick, MA) and the Psychtoolbox 3.0 package (Brainard, 1997; Kleiner, 2007) running on a standard Windows-based desktop computer.

### Experimental design and statistical analyses

#### BASE TASK

Every variant of SpotChase followed the same general sequence of events as the base version (Fig. 1a), which proceeded as follows. Data were collected in “runs,” each one lasting 90 seconds. A run began with the participant fixating on a red target at the top-center of the screen. Following fixation, two yellow targets (RGB vector [0.62 0.61 0], 1.5° in diameter) appeared at 8° to the left and right of the screen center. This was followed by a variable interval referred to as the stimulus onset asynchrony, or SOA (50–300 ms in increments of 50 ms). After the SOA elapsed, one yellow target, referred to as the cue, switched to either red (RGB vector [1 0.25 0.25]) or blue (RGB vector [0.25 0.55 1]). All stimuli were of high luminance (32 cd m^−2^). Thereafter, nothing changed until the participant made a saccade into one of the two invisible spatial boxes surrounding the cue and the noncue. Such boxes were rectangular, each centered on the corresponding target stimulus, and measured 7×10°, with the long side aligned with the choice axis (horizontal, in this case). Following a choice, i.e., a saccade into one of the target boxes, the display remained the same for an additional 150 ms. Then, simultaneously, the horizontal targets disappeared and two vertical yellow targets appeared at 8° up and down from the screen center. Thus, the choice cycle started anew, unfolding with the same sequence of events: targets on, cue, and choice saccade. Horizontal and vertical choices alternated thereafter throughout the duration of the run. Cue colors and locations were selected randomly.

The instructions given to participants were simple: to look at one of the two target stimuli present on the screen at any point in time, in order to maximize their score at the end of the run. Participants could make saccadic choices before or after the cue was revealed. The run score depended on the point values assigned to the three stimulus colors: 3 points were awarded for looking at a red target, 1 for looking at a yellow target, and 0 for looking at a blue target. To incentivize participants to make choices rapidly, even if that meant guessing on occasion, in a small fraction of the choices (10%) the cue was never shown (infinite SOA). In this case, participants had to select one of the two yellow targets at random. This got the participants accustomed to guessing and discouraged them from waiting too long for the cue. The actual score for each run was computed using the total accrued points divided by the total reaction time (RT) taken by all the choices, where the RT of an individual choice was measured from the onset of the yellow targets to the onset of the choice saccade (this made the score slightly more sensitive to the pace at which participants moved their eyes than the raw sum of points). The score depended on both the participant’s accuracy for discriminating colors and on their urgency, because given the fixed duration of each run (90 s), the less time spent pondering each choice, the more choices could be made.

#### TASK VARIANTS

To acclimate them to the task environment, participants performed a few short (30 s) practice runs, first with red cues only, then with blue cues only, and then with both interleaved. Participants required 3–5 such practice runs before beginning data collection with the base version of SpotChase.

All variants of SpotChase followed the same conventions as the base (Fig. 1a), but with alterations to the properties of the displayed stmuli. In the salient-noncue variant (SNC; Fig. 3a), the initial yellow targets were of low luminance (RGB vector [0.11 0.11 0]). Then, after the SOA, the noncue switched to a high-luminance yellow and was paired with either a low-luminance red (RGB vector [0.16 0 0]) or a low-luminance blue cue (RGB vector [0 0.03 0.23]). The (low) luminance was the same for the three dim colors (5 cd m^−2^), and the (high) luminance for the bright yellow was the same as for the base variant (32 cd m^−2^). In this case, the yellow, uninformative noncue was meant to be more physically salient than the red or blue cues, which still informed what the better choice was.

In the two spatial bias (SB) variants of SpotChase (Fig. 4a), SB1 and SB2, all stimulus colors and task conditions were the same as in the base version except that the cue locations were predictable. In the SB1 case, the cue always appeared on the top (in vertical choices) or on the left (in horizontal choices); whereas in the SB2 case, the cue always appeared on the bottom (in vertical choices) or on the right (in horizontal choices). For each of the participants who performed this version of the task, full datasets were collected sequentially and in the same order, first SB1 and then SB2. In this case, for each choice, the participants knew where the informative cue would appear, but still could not predict the location of the higher-value stimulus.

The pro-anti ratio (PAR) variant of SpotChase was the same as the base version except that, instead of the red and blue cues appearing with equal probabilities (1:1 red-to-blue ratio), one of them was more frequent. Participants who performed this version of the task experienced either predominantly red cues (3:1 or 6:1 ratio) or predominantly blue cues (1:3 or 1:6 ratio), with the probability ratio fixed for any given run. For this experiment, participants completed full datasets in a fixed sequence of ratios: 3:1, 6:1, 1:3, and 1:6. In this case, relative to cue salience, one of the two stimulus-response rules (look at the salient cue; look at the less salient noncue) was much more common than the other.

Finally, in the extreme-value (EV) variants of SpotChase, the stimuli were again the same as in the base condition but their assigned point values were different. In the EVB case, red, yellow, and blue stimuli were worth 1, 0, and −5 points, respectively, so the priority for participants was to avoid looking at any blue target. In the EVR case, red, yellow, and blue stimuli were worth 5, 0, and −1 points, respectively, so the priority for participants was to look at any red target. Participants were informed of the changes in point values and made aware that the run score would be maximized by prioritizing performance either on blue-cue choices (during EVB runs) or red-cue choices (during EVR runs). Participants first completed the EVB runs and then the EVR runs.

All 19 participants performed the base version of SpotChase. For the subsequent variants, they were randomly assigned to two groups. All members of group 1 (7 females, 2 males) performed the SNC variant, and thereafter, 8 of them (7 females, 1 male) also peformed the SB1 and SB2 variants. All members of group 2 (6 females, 4 males) performed the PAR and EV variants, in that order.

#### DATA PROCESSING

Individual saccades were detected offline using a velocity criterion (40°/s). As mentioned above, for each choice offered, the RT was computed as the interval between the onset of the yellow targets and the onset of the corresponding response saccade. Choices were excluded from analysis if they occurred before the onset of the yellow targets (which would produce RT < 0); if the RT was too long (> 800 ms); if a blink occurred within 100 ms of the response saccade onset; or if entry into a valid response box was registered but the velocity threshold was not met. Apart from these cases, which constituted approximately 14% of all detected choice saccades, no other choice saccades were excluded from analysis. Results were robust relative to changes in data inclusion/exclusion criteria (Fig. 2).

From each participant and each of the two main task conditions (red cue, blue cue), we collected a median of 1,122 choices (range: 584–1,431) suitable for analysis in the base version of the task. We aimed for this amount of data based on prior results indicating that this number was appropriate for resolving individual performance changes with a temporal resolution on the order of 10 ms or so (Shankar et al., 2011; Salinas et al., 2019; Goldstein et al, 2022, 2024). The numbers were similar for the other task variants, except for the ratio or PAR variant. In that case, we aimed to collect approximately the same amount of data (~1,100 choices) from the less frequent condition, so many more trials were collected from the complementary, high-frequency one. For example, for a pro-anti ratio of 3:1, the 1,100 anti choices were accompanied by approximately 3,300 pro choices.

Except for the very start of each run, no explicit fixation requirements were imposed. Participants sometimes made a saccade to the target opposite to the chosen one immediately after making an error, and sometimes made a saccade to the center of the screen before making an unequivocal choice. These movements, and others that did not bring the eyes into the boxes surrounding the two current valid targets, were not considered response saccades and were ignored. Critically, their existence was not detrimental to the measured relationship between choice accuracy and time: the PT was measured from the moment the color cue was revealed until the eyes reported an unambiguous choice, and the PT consistently dictated the probability of success regardless of whether intermediate (non-choice) saccades were more or less likely (Fig. 2b).

The PT or cue viewing time associated with each choice was equal to the time elapsed between the onset of the cue and the onset of the response saccade (which entered one of the target boxes). In practice, it was calculated as PT = RT − SOA, where all three quantities are specific to each choice. For analysis purposes, a choice was scored as ‘correct’ when the participant looked at the target with the higher assigned value given the alternatives, even if the corresponding saccade was initiated when both targets were still yellow (i.e., it was a guess).

#### DATA ANALYSIS

Data analyses were performed in the Matlab programming environment (The MathWorks, Natick, MA). As in the case of trial-based urgent tasks (Salinas et al., 2019; Poth, 2021; Goldstein et al., 2022, 2024; Oor et al., 2023, 2024), tachometric curves were produced by indexing trials by PT and sorting them into PT bins that shifted every 1 ms. The bin width was 81 ms for calculating the probability of capture (explained below) and 41 ms for all other analyses. The fraction of correct responses was then calculated for all the trials inside each bin. The resulting curve describes how choice accuracy depends on PT, or cue viewing time.

Whenever possible, analyses were carried out both for individual participants and for their pooled data. Pooling means that all the choices from a given group of participants were combined into one large dataset, as if produced by a single, aggregate participant.

For some analyses, performance was quantified as the mean fraction of correct choices over a wide PT window. For the SB variant, choice biases were measured via the fraction correct for guesses (PT < 90 ms). For the SNC variant, we considered the fraction correct in an intermediate window where exogenous capture was most likely (80 < PT < 170 ms). For the PAR variant, we considered the mean fraction correct at a long-PT window for which choices were most likely informed by the cue (170 < PT < 350 ms). For any given fraction correct, a 95% or 68% confidence interval (CI) was calculated using binomial statistics; specifically, the Agresti-Coull method (Agresti and Coull, 1998).

To quantify the magnitude of exogenous capture, we measured the fraction correct within a fixed PT window, as described in the previous paragraph, and we also calculated the probability of capture, or $P_{CAP}$, which does not require a predetermined PT window. It was defined as

(1) $$P_{CAP}=\frac{X}{X\;+\;Y}$$

where X represents the area between the tachometric curve and the chance line when the curve is below said line, and Y is the area between the curve and the chance line when the curve is above. This quantity corresponds to the proportion of non-random choices that were erroneous: of those saccades that were not guesses, $P_{CAP}$ is the fraction that were directed toward the worse alternative. Thus, $P_{CAP}$ is one (zero) when performance is below (above or at) chance for the full PT range considered. Shown results are based on $P_{CAP}$ as computed directly from the empirical tachometric curves in the range 70–400 ms. An alternative calculation of $P_{CAP}$, also defined via Equation 1 but derived from analytical fits to the tachometric curves, produced qualitatively similar results. CIs for $P_{CAP}$ were obtained by bootstrapping (see below).

We also designed a randomization test specifically to evaluate the significance of each $P_{CAP}$ value, i.e., the probability that a positive value was obtained just by chance when, in fact, performance was never truly below 50% correct. To to this end, we found all the PT bins where the value of the tachometric curve dipped below 50% correct and substituted the empirical ordinate values with random binomial samples (also below 50% correct) based on the same numbers of trials contained in each PT bin and assuming that the true probability correct was 50%. Then $P_{CAP}$ was recomputed from the resulting (resampled) tachometric curve. This resampling process was repeated 10,000 times to obtain a distribution consistent with the (null) hypothesis that the performance curve dipped below chance only because of random fluctutations due to limited sampling. The significance p-value was equal to the fraction of resampled $P_{CAP}$ values that was equal to or larger than that obtained empirically; it had a precision of 0.0001, given the number of iterations used.

As in prior studies (Shankar et al., 2011; Salinas et al., 2019; Goldstein et al., 2022, 2024), the empirical tachometric curves were fitted with continous analytical functions to extract key performance parameters. Tachometric curves that increased monotonically (e.g., for red-cue choices in the base task) were fitted with an increasing sigmoid function defined as

(2) $$s(x)=B+\frac{A\;-\;B}{1\;+\;exp\left(-\frac{x-C}{D}\right)}$$

where D determines the slope of the curve; B and A represent chance and asymptotic accuracy levels, respectively; and C is the PT at which the fraction correct is halfway between B and A.

Tachometric curves that showed a substantial dip below chance (e.g., during blue-cue choices in the base task) were fitted using a combination of two sigmoidal curves, as previously described (Salinas et al., 2019; Goldstein et al., 2022). The fitting curve v(x) was defined as

(3) $$v(x)=max\left(s_{L}(x),s_{R}(x),0\right)$$

where the function max(a,b,c) returns the largest of a, b, or c. Here, the left and right sigmoids are given by

(4) $$s_{L}(x)=B_{LR}+\frac{A_{L}\;-\;B_{LR}}{1\;+\;exp\left(\frac{x-C_{L}}{D_{L}}\right)}$$

(5) $$s_{R}(x)=B_{LR}+\frac{A_{R}\;-\;B_{LR}}{1\;+\;exp\left(-\frac{x-C_{R}}{D_{R}}\right)}$$

where the left, decreasing side of the tachometric curve is tracked by $s_{L}$ and the right, increasing side is tracked by $s_{R}$. Here, $A_{L}$ represents chance performance (at short PTs), $B_{LR}$ represents minimum performance (below chance), and $C_{L}$ is the halfway point between the two. Similarly, $A_{R}$ represents asymptotic accuracy (at long PTs), and $C_{R}$ denotes the halfway point between the minimum $\left(B_{LR}\right)$ and asymptotic $\left(A_{R}\right)$ levels.

The main quantity derived from the analytical fits was the rise point, which was defined as the PT at which the fitting curve first exceeds a threshold value of 70% correct. By using such a fixed performance criterion, the timing of pro (red-cue) and anti (blue-cue) choices could be compared most directly (black points in Fig. 1b–d). In addition, as mentioned above, the same fits were used to compute $P_{CAP}$ in an alternative way. For this, Equation 1 was applied to each fitting curve.

Tachometric curves were fitted to Equations 2–5 by finding parameter values (A,B,C, etc) that minimized the mean absolute error between the empirical data and the corresponding analytical curves. The Matlab function fminsearch was used for this optimization step. As done previously (Salinas et al., 2019), CIs were calculated for the fit parameters, as well as for any quantities derived from the fits (rise point, $P_{CAP}$), by bootstrapping (Davison and Hinkley, 2006; Hesterberg, 2014). That is, the trial-wise data were resampled with replacement, the resulting (resampled) tachometric curves were re-fitted, the new parameter values and derived quantities were saved, and the process was repeated many times (1000–10,000 iterations) to generate distributions for all the parameters and derived quantities. Finally, 95% CIs were obtained by calculating the 2.5 and 97.5 percentiles derived from the bootstrapped distributions.

Although the reported rise points were typically obtained via curve fitting, as described above, we also implemented a second method for computing them. It served both as a check on the fits and as an alternative for cases in which the tachometric curves were too noisy for reliable fitting. This alternative method operates on the empirical tachometric curve and consists of two steps. First, find any PTs where the tachometric curve is near 70% correct (say, between 65% and 75%). Then, from those PTs, select the largest, continuous PT interval for which (1) all the curve values are within the specified accuracy range, and (2) the curve values bordering the left and right edges of the interval are below and above the 65% and 75% limits, respectively. The rise point is then equal to the midpoint of the resulting PT interval. As with all other quantities, CIs were generated by bootstrap. This method produced very similar rise points as that based on curve fitting. It was used primarily for the history analyses, for which data were typically parsed into small subsets.

To compare different datasets where each data point represents an experimental result from one participant, significance was determined using permutation tests for paired data (Siegel and Castellan, 1988) or equivalent randomization tests for non-paired data. To compare groups involving binary data (correct vs incorrect), confidence intervals and significance values were determined using binomial statistics (Agresti and Coull, 1988).

#### DATA SORTING FOR HISTORY ANALYSIS

History-conditioned tachometric curves (Figs. 8, 9) were generated by only considering choices that were preceded by specific cue-color sequences. For this, choices were sorted post hoc according to the number of consecutive occurrences in which the cue color on immediately preceding choices was either the same (S) or different (D) from that on the current choice.

Choices classified as S were those preceded by N choices with a cue of the same color (e.g., Fig. 8a, b, e, yellow spectrum). The classification was such that choices preceded by at least 1 choice with a cue of the same color were termed 1S (for pro choices, red-**red** sequences; for anti choices, blue-**blue** sequences, where the bolded word indicates the choice of interest). Choices preceded by at least 2 choices with a cue of the same color were termed 2S (for pro choices, redred-**red** sequences; for anti choices, blue-blue-**blue** sequences), etc. The S choices reveal the impact of color repetition.

Analogously, choices classified as D (e.g., Fig. 8a, b, e, red spectrum) were those preceded by N choices with a cue of the alternative color, which was different. As such, choices preceded by at least 1 choice with a cue of the opposite color were designated 1D (for pro choices, blue-**red** sequences; for anti choices, red-**blue** sequences). Choices preceded by at least 2 choices with a cue of the opposite color were designated as 2D (for pro choices, blue-blue-**red** sequences; for anti choices, red-red-**blue** sequences), and so on. The D choices reveal the impact of a switch in cue color.

### Sources

All content was generated by humans.

## Abbreviations:

CI: confidence interval
EV: extreme-value
PAR: pro-anti ratio
$P_{CAP}$,: probability of capture
PT: processing time
RT: reaction time
SB: spatial bias
SNC: salient noncue
SOA: stimulus onset asynchrony
